# Analysis of hemodynamics and impedance using bioelectrical impedance analysis in hypovolemic shock-induced swine model

**DOI:** 10.1038/s41598-024-65847-y

**Published:** 2024-07-02

**Authors:** Hoonsung Park, Hanyoung Lee, Seungmin Baik, Jae-Myeong Lee

**Affiliations:** 1 Division of Acute Care Surgery, Department of Surgery, Korea University Anam Hospital, Korea University Medical Center, Seoul, Republic of Korea; 2 Division of Critical Care Medicine, Department of Surgery, Ewha Womans University Mokdong Hospital, Ewha Womans University College of Medicine, Seoul, Republic of Korea

**Keywords:** Hypovolemic shock, Bioelectrical impedance, Resuscitation, Crystalloid, Albumin, Outcomes research, Experimental models of disease

## Abstract

To treat hypovolemic shock, fluid infusion or blood transfusion is essential to address insufficient volume. Much controversy surrounds resuscitation in hypovolemic shock. We aimed to identify the ideal fluid combination for treating hypovolemic shock-induced swine model, analyzing bioelectrical impedance and hemodynamics. Fifteen female three-way crossbred pigs were divided into three different groups. The three resuscitation fluids were (1) balanced crystalloid, (2) balanced crystalloid + 5% dextrose water, and (3) balanced crystalloid + 20% albumin. The experiment was divided into three phases and conducted sequentially: (1) controlled hemorrhage (1 L bleeding, 60 min), (2) resuscitation phase 1 (1 L fluid infusion, 60 min), and (3) resuscitation phase 2 (1 L fluid infusion, 60 min). Bioelectrical impedance analysis was implemented with a segmental multifrequency bioelectrical impedance analyzer. A total of 61 impedance measurements were assessed for each pig at six different frequencies in five segments of the pig. Pulse rate (PR), mean arterial pressure (MAP), stroke volume (SV), and stroke volume variation (SVV) were measured using a minimally invasive hemodynamic monitoring device. The three-dimensional graph showed a curved pattern when infused with 1 L of balanced crystalloid + 1 L of 5% dextrose water and 1.6 L of balanced crystalloid + 400 ml of 20% albumin. The 1M impedance increased in all groups during the controlled hemorrhage, and continuously decreased from fluid infusion to the end of the experiment. Only balanced crystalloid + 20% albumin significantly restored MAP and SV to the same level as the start of the experiment after the end of fluid infusion. There were no significant differences in MAP and SV from the time of recovery to the initial value of 1M impedance to the end of fluid infusion in all groups. The change and the recovery of hemodynamic indices such as MAP and SV coincide with the change and the recovery of 1M impedance. Using balanced crystalloid mixed with 20% albumin in hypovolemic shock-induced swine model may be helpful in securing hemodynamic stability, compared with balanced crystalloid single administration.

## Introduction

### Resuscitation of hypovolemic shock and the fluids of choice

Most hypovolemic shocks are due to hemorrhage. In clinical practice, they mainly appear as traumatic hemorrhagic shocks^1^. Damage control surgery to obtain early bleeding control and prevent infection from intraabdominal injuries has long been considered the initial treatment for hemorrhagic shock. Recently, for advances in basic and clinical research, the concept of damage control resuscitation (DCR) has emerged^2,3^. Resuscitation means restoration of circulation and perfusion. Generally, resuscitation from shock means restoration of organ perfusion^4^.

Much controversy surrounds resuscitation in hypovolemic shock. To treat hypovolemic shock, fluid infusion or blood transfusion is essential to address insufficient volume. DCR comprises (1) minimized administration of isotonic crystalloid, (2) permissive hypotension, (3) balanced ratio transfusion, and (4) goal-directed correction of coagulopathy^5^. In hypovolemic shock, administration of blood products is the most ideal fluid of choice. However, unless for an emergency transfusion using unmatched type-O packed red blood cells, blood type testing and antibody screening take time, and are costly. Therefore, initial resuscitation treatment for patients with hypovolemic shock is generally implemented with intravenous fluids, especially crystalloids.

Intravenous fluids are categorized as crystalloid and colloid. Much research and many controversies surround the choice of the fluids and how much is the most effective amount of the fluids in patients with shock. In vitro studies have reported that hypertonic solutions reduce the activity and adhesion of neutrophils, promote proliferation of lymphocytes, inhibit pro-inflammatory responses, and promote the secretion of anti-inflammatory cytokines secreted by monocytes and macrophages^6^. These results have been spotlighted because they have an immunomodulatory effect on leukocyte activation induced by bleeding and followed by resuscitation. However, in clinical trials, hypertonic saline and hypertonic dextran led to a higher early mortality rate with no difference in mortality rate between the experimental and control groups^7^. In the past, fluid treatment using colloid received attention. However, in 2013, hydroxyethyl starch (HES), a synthetic colloid, was recommended for suspension by the European Medicine Agency (EMA) and Pharmacovigilance Risk Assessment Committee (PRAC) for critically ill patients with sepsis, burns, or trauma. Moreover, in 2018, the suspension of HES was totally confirmed by EMA and PRAC^8,9^. Thus, it has been used limitedly in acute bleeding patients. However, in 2022, the suspension of HES was confirmed again, even for use in cases of acute blood loss^10^. In 2013, the United States Food and Drug Administration recommended prohibiting the use of HES in patients with sepsis, severe liver disease, coagulation disorders due to underlying diseases, and critically ill adults^11^. Gelatin, dextran, albumin, and isotonic crystalloid can be considered substitutes for HES in fluid treatment^12^. Among colloids, albumin is regarded as an appropriate fluid of choice.

The common denominator of shock is endothelial barrier dysfunction^13,14^. Although the mechanisms of septic and traumatic shock are different, they commonly result in the loss of integrity of the endothelial cell barrier that leads to the activation of endothelial cells^15^. The current treatment strategy for traumatic hypovolemic shock is an early whole blood transfusion or transfusion of red blood cells, plasma, and platelets in a balanced ratio^16,17^. Excessive infusion of crystalloid in patients with traumatic hemorrhagic shock causes increased mortality, increased endothelial cell permeability, inflammatory response, and decreased perfusion of vital organs^18–20^. Fluids have various effects on the integrity of endothelial cells. Fluids with low protein content deteriorate glycocalyx shedding. Glycocalyx is a protein and polysaccharide substance attached to the cell membrane and is involved in cellular integrity. Plasma, a protein-rich fluid, is thought to be superior to normal saline in protecting the glycocalyx and endothelial cell barrier function. In a study on hemorrhagic shock-induced rodents, resuscitation with 5% albumin resulted in restoration of the thickness of the glycocalyx and less shedding of syndecan-1 compared with crystalloids^21^. This suggests that albumin may be helpful in resuscitation during hypovolemic shock.

Albumin is a fluid that can obtain similar intravascular volume in small amounts compared with crystalloids. Additionally, volume expansion of up to 300–500% is possible with 25% albumin^22^. Several animal studies have shown that albumin has protective effects against lung injury during resuscitation for acute hypovolemic/hemorrhagic shock^23^. However, several large-scale, multicenter, randomized controlled trials have shown that albumin did not show superior results compared with other fluids^24–28^. Additionally, in critically ill patients with traumatic brain injury, resuscitation with albumin was associated with a higher mortality rate^29^.

### Monitoring the resuscitation of hypovolemic shock: the FloTrac system and bioelectrical impedance analysis

In the resuscitation of hypovolemic shock, it is important to determine which fluid and how much of it to administer, and evaluate the appropriateness of resuscitation. In clinical practice, hemodynamics are monitored by various methods: invasive Swan-Gantz catheter, esophageal Doppler, minimally invasive FloTrac system, and noninvasive bioelectrical impedance analysis (BIA). A device that uses the principles of BIA is impedance cardiography, which monitors hemodynamics by analyzing the electrical impedance of the chest wall^30^. BIA has some advantages: it is noninvasive, can be performed at the bedside, and is capable of repeated measurements.

One study conducted body water measurement through BIA in septic shock patients. In the non-survivor group, the ratio of extracellular water volume to total body fluid volume significantly increased 1 h after fluid treatment, while the ratio of intracellular water volume to total body fluid volume decreased^31^. However, there has been no analysis of impedance change using BIA in a hypovolemic shock swine model, or impedance change according to resuscitation fluid.

When hypovolemic shock occurs, extracellular and intracellular water is lost. When intracellular dehydration occurs, the capacity of the cell membrane is decreased, which deteriorates its integrity and function^32^. In critically ill patients, cell shrinkage followed by cellular dehydration is hypothesized to lead to significant loss of body proteins^33^. Among crystalloids, glucose of 5% dextrose water is metabolized in the body and then functions as free water, which helps supplement intracellular water. Therefore, a fluid that can replenish intracellular water should help prevent cell shrinkage and functional deterioration that can occur during hypovolemic shock.

In this study, impedance changes according to the type of resuscitation fluid in a hypovolemic shock-induced swine model were measured using a BIA device. The resuscitation fluids were divided into three groups: (1) balanced crystalloid, (2) balanced crystalloid + 5% dextrose water, and (3) balanced crystalloid + 20% albumin. We aimed to identify the ideal fluid combination for treating hypovolemic shock by analyzing impedance change and hemodynamics.

## Materials and method

### Animal preparation

Fifteen female three-way crossbred pigs (Yorkshire × Landrace × Duroc) were used. Their average age was 89.7 ± 6.4 days (distribution: 80–100 days), and average weight was 37.7 ± 3.2 kg (distribution: 34.2–41.5 kg). Experiments were performed according to the Guide for the Care and Use of Laboratory Animals and Animal Research: Reporting of In Vivo Experiments (ARRIVE) guidelines. A seven-day quarantine and acclimatization period was provided before the experiment. In a space where temperature and humidity were controlled, water was provided without restrictions, and feed was provided once a day. A fasting period of 24 h was maintained before the experiment. Randomization was carried out as follows. On arrival, animals were assigned a group designation. A total number of 15 animals were divided into three different groups (five animals per group). Each animal was assigned a temporary random number. For each animal, three different investigators were involved as follows: a first investigator (JML) administered the treatment based on the randomization table. This investigator was the only person aware of the treatment group allocation. A second investigator (DHL, a veterinarian of our center) was responsible for the anaesthetic procedure, whereas a third investigator (HP) performed the surgical procedure.

### Preparation for implementation of hypovolemic shock model

#### Anesthesia and catheter insertion

The experimental animals were sedated with intramuscular Tiletamine-Zolazepam (5 mg/kg). After orotracheal intubation, anesthesia induction and maintenance were implemented with intramuscular xylazine (1–2 mg/kg), isoflurane 2%, and oxygen 50%. The lungs were mechanically ventilated by an anesthesia machine (Mindray WATO-EX35; Shenzhen Mindray Bio-medical Electronics Co. Ltd.), and the tidal volume was 12–15 mg/kg and respiratory rate was 12–15 times/min in volume-controlled mode. The experimental animals were placed in a supine position with their limbs fixed on an operation table, and the table was set at 37 °C to maintain body temperature. A 7-French three-lumen central venous catheter (Edwards Life Sciences, Irvine, CA, USA and Arrow International, Inc., Reading, PA, USA) was inserted into the right internal jugular vein, and an arterial catheter was inserted into the left femoral artery. A 5-French Angio catheter for bleeding was inserted into the right femoral artery. An 8-French foley catheter was inserted into the bladder.

#### Monitoring

Pulse rate (PR), mean arterial pressure (MAP), stroke volume (SV), and stroke volume variation (SVV) were measured using a minimally invasive hemodynamic monitoring device (FloTrac™, Edwards Lifesciences, Irvine, CA, USA). When the blood pressure was too low to detect MAP because of the bleeding that did not display its waveform on the monitor, MAP, SV, and SVV could not be measured. Urine output was measured every hour. Vital signs were checked every 1 min, and BIA was performed every 3 min for a total of 180 min.

#### Experiment protocols

**Figure 1:**
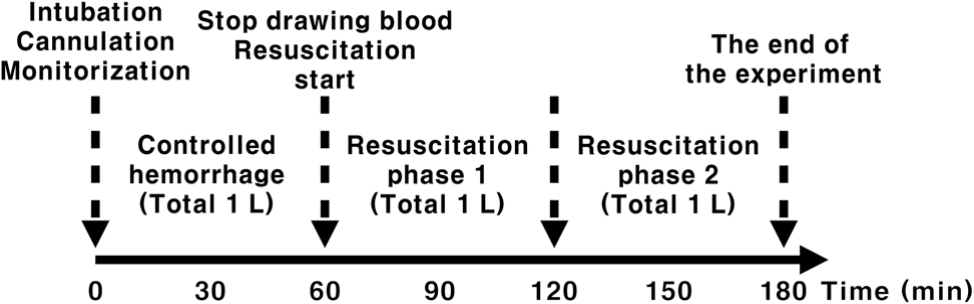
Experimental process.

The experiment was divided into three phases: (1) controlled hemorrhage (1 L bleeding, 60 min), (2) resuscitation phase 1 (1 L fluid infusion, 60 min), and (3) resuscitation phase 2 (1 L fluid infusion, 60 min) (Fig. 1). Controlled hemorrhage was implemented by connecting a 3-way cock to a catheter inserted into the right femoral artery, and 100 ml of blood was withdrawn every 6 min 10 times, for a total of 60 min. A fixed volume hemorrhage protocol was adopted. Hemorrhagic shock in pigs is induced when there is a blood loss of 30–65% of the estimated blood volume (58–74 ml/kg)^34^. The body weight of the pigs and 1 L of controlled hemorrhage used in this study are within the range that causes hemorrhagic shock by this definition above. The data on average proportion of blood loss per body weight or body surface area is provided in Supplement 1.

Resuscitation fluids were injected according to the protocol through a central venous line. Resuscitation phases 1 and 2 were conducted sequentially. A total of 2 L of fluid was administered to each pig for resuscitation. The three combinations of resuscitation fluids are presented in Table 1. Fifteen pigs were evenly divided into three groups according to the combination of resuscitation fluids. The balanced crystalloid solution used was Plaju OP inj. (JW Choongwae Pharmaceutical Co., Ltd.). Additionally, 5% dextrose water (Daihan Pharmaceutical Co., Ltd.) and 20% albumin (GC Biopharma Co., Ltd.) were used for administration.

**Table 1:** Combination and amount of fluids according to the experimental phase.

	Group 1	Group 2	Group 3
Resuscitation, phase 1	Balanced crystalloid 1 L	Balanced crystalloid 0.5 L + 5% dextrose water 0.5 L	Balanced crystalloid 0.8 L + 20% albumin 200 ml
Resuscitation, phase 2	Balanced crystalloid 1 L	Balanced crystalloid 0.5 L + 5% dextrose water 0.5 L	Balanced crystalloid 0.8 L + 20% albumin 200 ml
Total amount	Balanced crystalloid 2 L	Balanced crystalloid 1 L + 5% dextrose water 1 L	Balanced crystalloid 1.6 L + 20% albumin 400 ml

#### Bioelectrical impedance analysis (BIA)

**Figure 2:**
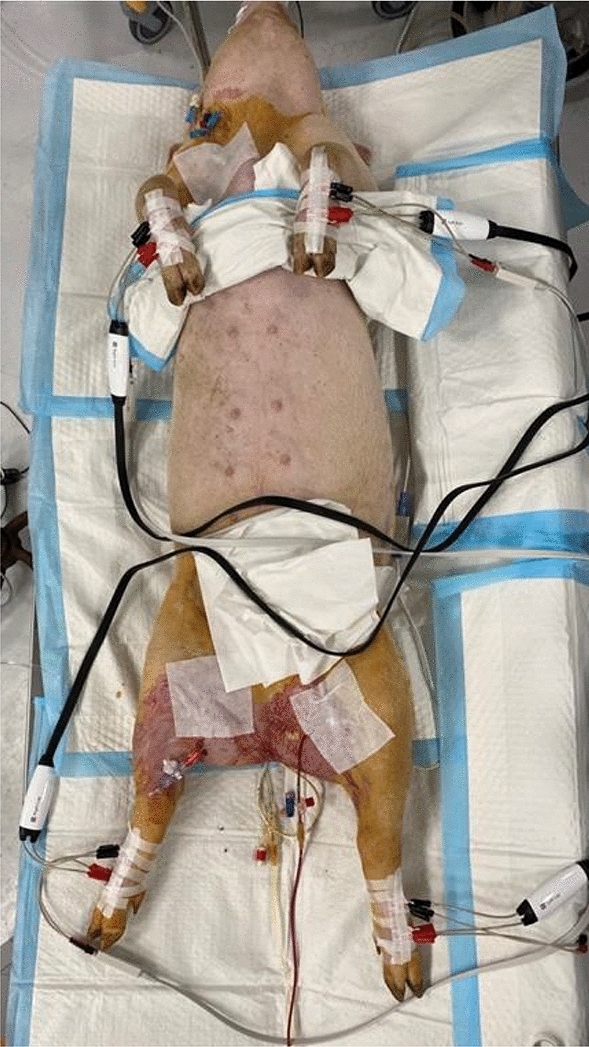
BWA 2.0, the segmental multifrequency BIA is attached.

BIA was implemented with BWA 2.0 (Inbody co., Seoul, Korea), a segmental multifrequency bioelectrical impedance analyzer (Fig. 2). Segmental multifrequency BIA divides the body into five cylinders except the head; thus, it can accurately measure impedance compared with whole-body BIA, which recognizes the body as one cylinder^35^.

Eight-point electrodes were used, with four electrodes attached to each foot and four to each limb. A total of 61 impedance measurements were assessed for each pig at six different frequencies (1 kHz, 5 kHz, 50 kHz, 250 kHz, 500 kHz, 1000 kHz) in five segments of the pig (right arm, left arm, torso, right leg, and left leg). Before starting the experiment, BIA was performed three times to confirm the baseline and check for nonspecific noise.

BIA measures the impedance (Z) by flowing an electric current through the human body. Impedance (Z) is the vector sum of resistance (R) and reactance (X)^36^. Low- and high-frequency currents are used to measure impedance in BIA devices. The low-frequency current does not penetrate the cell membrane. Therefore, only the impedance of extracellular water can be measured. The high-frequency current penetrates the cell. Therefore, the impedance of both intracellular and extracellular water can be measured. In this experiment, the low-frequency current of 5 kHz and high-frequency current of 1000 kHz (1 MHz) were used^37^.

### Principle of BIA and analysis of the three-dimensional graph of the time-impedance-impedance ratio

In contrast to traditional BIA, which recognizes the subject’s body as a single cylinder, multifrequency BIA divides the whole body into five segments and measures body water. The human body is composed of water that conducts electric current well. Resistance is the degree to which an electric current passes through and varies depending on the amount of water. Based on this principle, body water is measured by calculating the impedance that occurs when a small alternating current flows through the human body. The cell membrane separates extracellular and intracellular water in cells. This cell membrane functions as a capacitor. At low frequencies, an electric current cannot pass through cell membranes, but it can do so at high frequencies. Therefore, extracellular water can be measured at low frequencies, and total body water can be measured at high frequencies^38^. As controlled hemorrhage continues, total body water decreases, leading to an increase of impedance. As fluid infusion continues, total body water increases leading to a decrease of impedance. As 5K impedance is a low-frequency impedance and reflects only extracellular water, the change in total body water amount due to controlled hemorrhage and fluid infusion in pigs can be checked by measuring 1M impedance, which is a high-frequency impedance. As the torso is expected to have the most blood reserves among the limbs and torso of the pigs, impedance values of the torso were used for analysis.

Impedance and impedance ratio over time alone are not sufficient to determine which fluid combinations contribute to the increase in intracellular water. If the two-dimensional graphs of time-impedance and time-impedance ratio are expressed as the 3D graphs of time-impedance-impedance ratio, it is easy to identify the fluid combinations affecting intracellular water. The 3D graph sets the X axis as low-frequency impedance, Y axis as low-frequency impedance/high-frequency impedance, and the time as colors. If the graph shows a linear pattern, Z_in_ (impedance of intracellular water) will not be changed as a constant. If the graph shows a curved rather than a linear pattern, Z_in_ (impedance of intracellular water) will be changed as a variable (Eq. 1). This 3D graph can measure changes in intracellular water over time during controlled hemorrhage and fluid infusion. As mentioned in the Data Analysis section, as the impedance values for each group were standardized, it is possible to check changes in intracellular water through changes in the graph and compare the results.

1 $$\begin{gathered} \begin{array}{*{20}l} {{\text{Z}}_{{{\text{ex}}}} } & {\frac{{{\text{Z}}_{{{\text{in}}}} {\text{ + Z}}_{{{\text{ex}}}} }}{{{\text{Z}}_{{{\text{in}}}} }}} \\ {\text{X = low frequency impedance (extracellular water)}} & {{\text{Y = low frequency impedance (5K)/high frequence impedance (1M) (the ratio of extracellular water/intracellular water)}}} \\ \end{array} \hfill \\ {\text{Equation 1. Variables for the three dimensional graph of the time-impedance-impedance ratio}} \hfill \\ \end{gathered}$$

### Data analysis

We analyzed temporal changes of impedances and vital signs during controlled hemorrhage and fluid infusion. The impedance value was standardized to minimize differences in impedance changes among the body parts, frequencies, and pigs. Among the five-segment impedance values that can be measured using BWA 2.0, only the impedance of the torso was analyzed because of the possible inaccuracy of the impedance measurement of the four limbs. The Z-score method was used to equally calibrate impedance changes of different absolute value changes over time to the same scale. Standardization was performed so that the overall average was always 0. Group-level median curves over time were calculated, with normalized medians from standardized data, to reduce inter-pig differences in impedance changes following controlled hemorrhage and fluid infusion. The median value was taken as the top value of the probability distribution produced by five pigs using the maximum likelihood estimation.

A three-dimensional (3D) matrix can be created when we collect the data as “5 parts (right arm, left arm, torso, right leg, left leg) × 2 frequencies (5k, 1M) × 5 pigs (number of pigs per fluid combinations).” To standardize and calibrate the changes in data from each pig, B-SPLINE data interpolation at the 25th percentile, 50th percentile, and 75th percentile points was used. Additionally, the initial value was set as the baseline and 0, which is the unified starting point of the impedance change.

### Statistical analysis

Statistical analysis was conducted using MATLAB (version 2021a, Mathworks Inc., Co., Ltd., USA) and SPSS (version 24, IBM Co., Armonk, NY, USA). To calculate the median value of a data group in impedance analysis, maximum likelihood estimation was conducted using the mle function provided in Toolbox, and the median value of the group distribution was extracted. To calculate percentile, the prctile function provided by Mathworks Inc. was used to extract the 75% and 25% percentile values. As there were few data at each time point (5), when using the prctile function, 25% and 75% percentile values were calculated using T-Digests based on linear interpolation. In analyzing the effect of the resuscitation fluids, PR, MAP, SV, and SVV of five pigs in each group at the five points of time were analyzed. The Jarque–Bera test of normality was conducted for the collected data. Then an independent sample *t*-test was conducted for the data at five points, by point to point, within a 95% confidence interval.

### Ethics

This experimental protocol was approved by the Institutional Animal Care and Use Committee of Korea University College of Medicine (approval number KOREA-2018-0129-C3) and performed in accordance with the guidelines for the care and use of laboratory animals^39^ and ARRIVE guidelines. Every effort was made to reduce the suffering of experimental animals.

## Results

**Figure 3:**
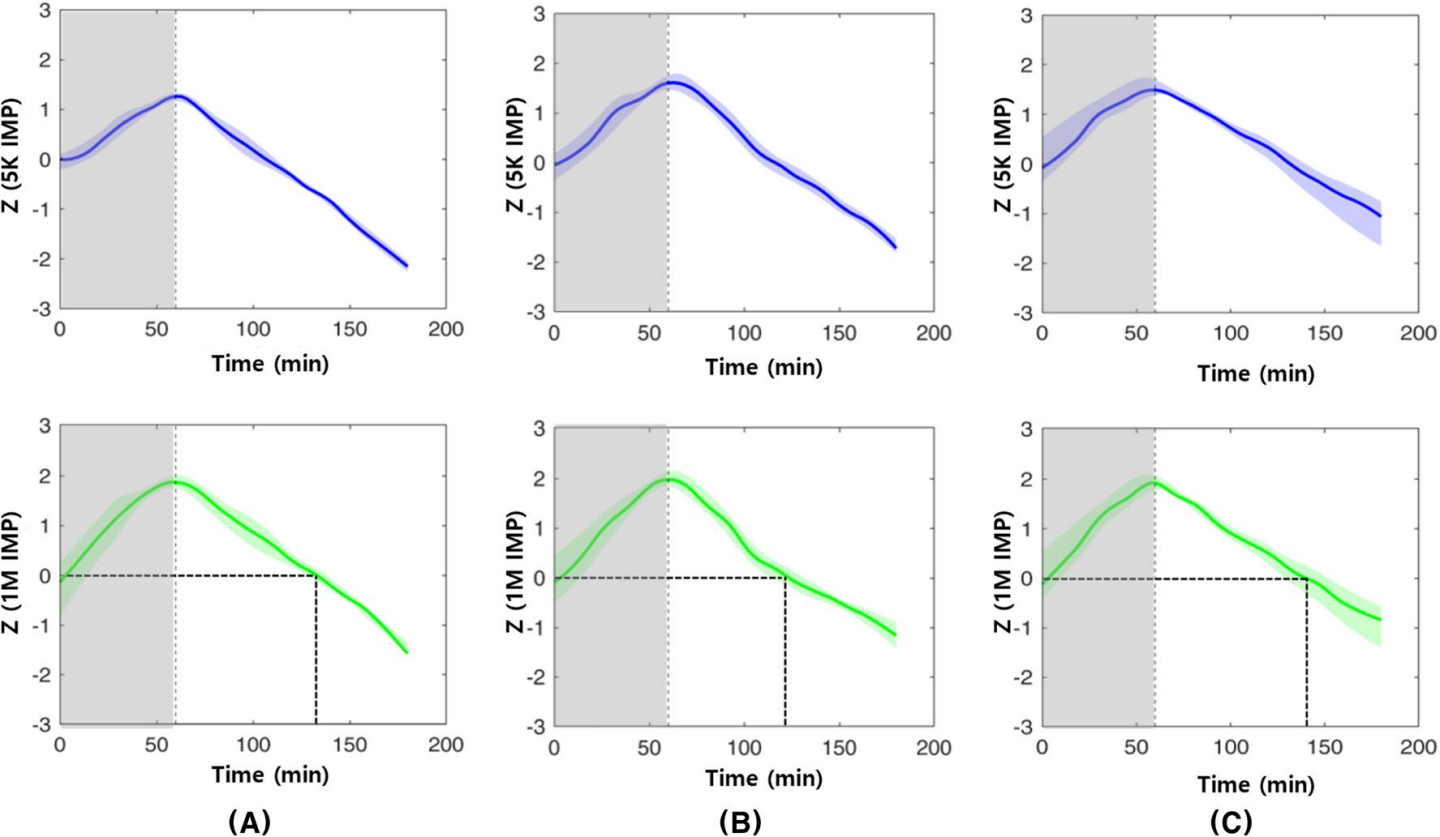
Impedances of the torso in the three groups: (**A**) balanced crystalloid; (**B**) balanced crystalloid + 5% dextrose water; (**C**) balanced crystalloid + 20% albumin, graphs of standardized median values. (Blue line – 5k impedance, green line – 1M impedance, the upper and lower part of the shaded region in each graph indicate 75th percentile and 25th percentile value, respectively).

Figure 3 shows the torso impedance results in the three groups. During controlled hemorrhage, impedance increased at both low (5 kHz) and high (1 MHz) frequencies (shaded area for the first 60 min in Fig. 3). After fluid infusion, impedance decreased at all frequencies (after 60 min in Fig. 3).

**Figure 4:**
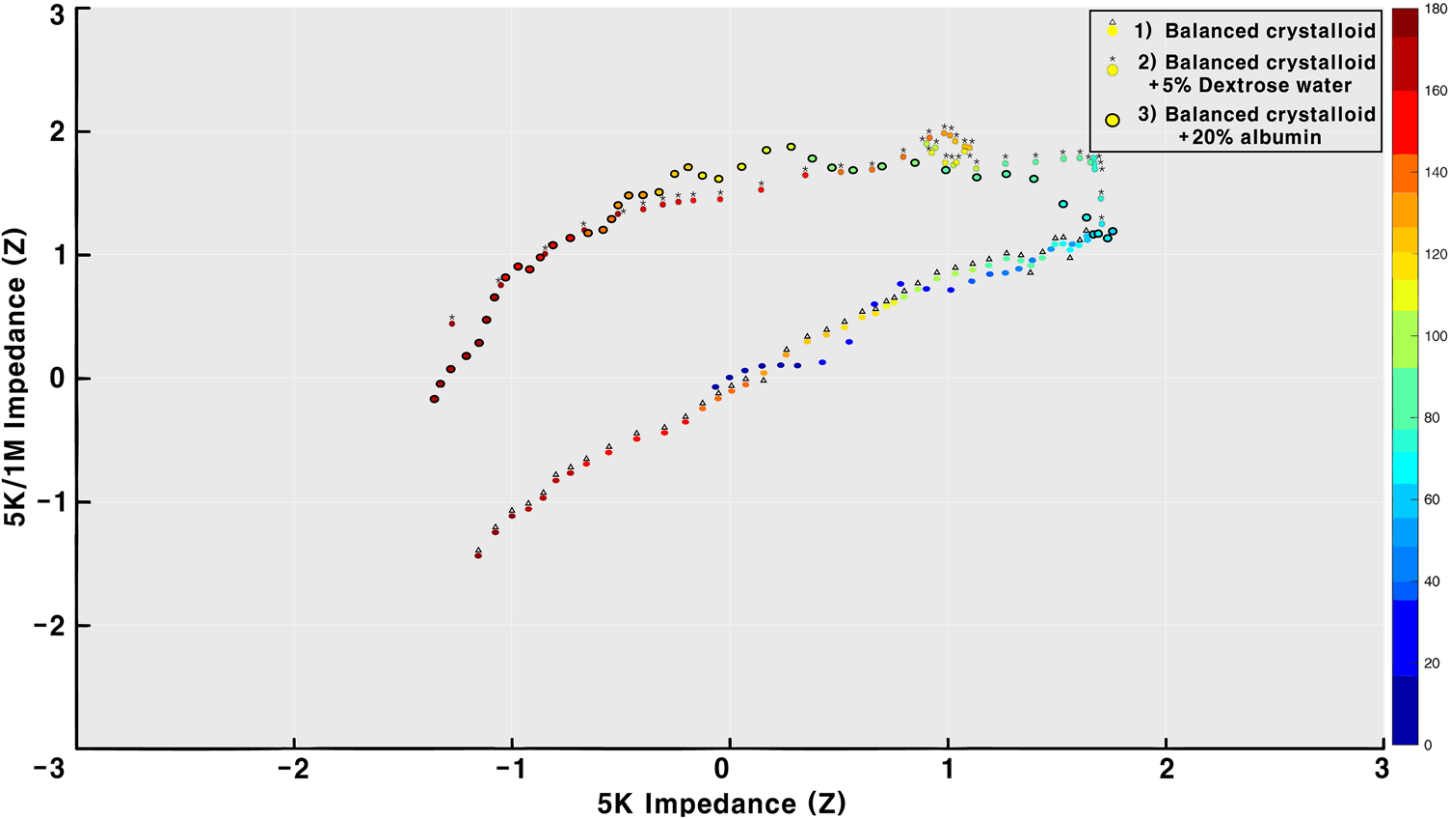
Three-dimensional time-impedance-impedance ratio graph. X = low-frequency impedance, Y = low-frequency impedance/high-frequency impedance, and the time is expressed in colors. The values of the X and Y axes are standardized (z-score). (1) Balanced crystalloid; (2) Balanced crystalloid + 5% Dextrose Water; (3) Balanced crystalloid + 20% albumin.

In the group infused with 2 L of balanced crystalloid, the 3D graph of the time-impedance-impedance ratio exhibited a linear change when fluid was infused after controlled hemorrhage (Fig. 4–1). In the group infused with 1 L of balanced crystalloid + 1 L of 5% dextrose water, the 3D graph of the time-impedance-impedance ratio exhibited a curved pattern rather than a linear one when fluid was infused after controlled hemorrhage (Fig. 4–2). The group infused with 1.6 L of balanced crystalloid + 400 ml of 20% albumin also exhibited a curved rather than a linear pattern in the 3D time-impedance-impedance ratio graph when infusing fluid after controlled hemorrhage (Fig. 4–3).

**Figure 5:**
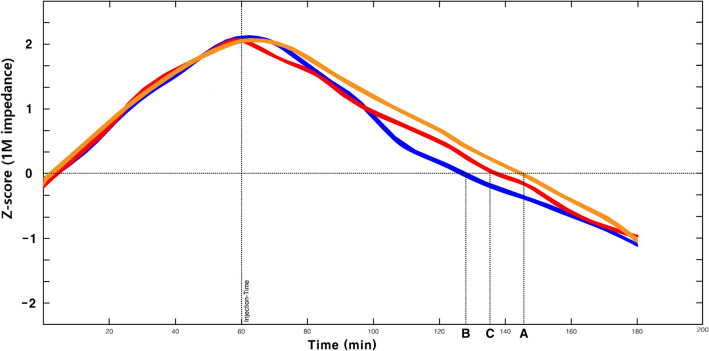
Changes of 1M impedance over time according to the three types of fluid combinations. X = Time (minutes), Y = Standardized value of 1M impedance (yellow line – balanced crystalloid, blue line - balanced crystalloid + 5% dextrose water, red line – balanced crystalloid + 20% albumin). The time of recovery to the initial value of 1M impedance to the end of fluid infusion are marked as: (A) balanced crystalloid, 146 minutes; (B) balanced crystalloid + 5% dextrose water; 128 minutes; (C) balanced crystalloid + 20% albumin, 135 minutes.

The time-dependent changes in 1M impedance, which reflects the total body water for each group, are shown in Fig. 5. After the start of the experiment, the 1M impedance increased in all three groups during the controlled hemorrhage, and continuously decreased after fluid infusion until the end of the experiment. The recovery time for the 1M impedance value to the start of the experiment was 146 min for balanced crystalloid, 128 min for balanced crystalloid + 5% dextrose water, and 135 min for balanced crystalloid + 20% albumin.

**Figure 6:**
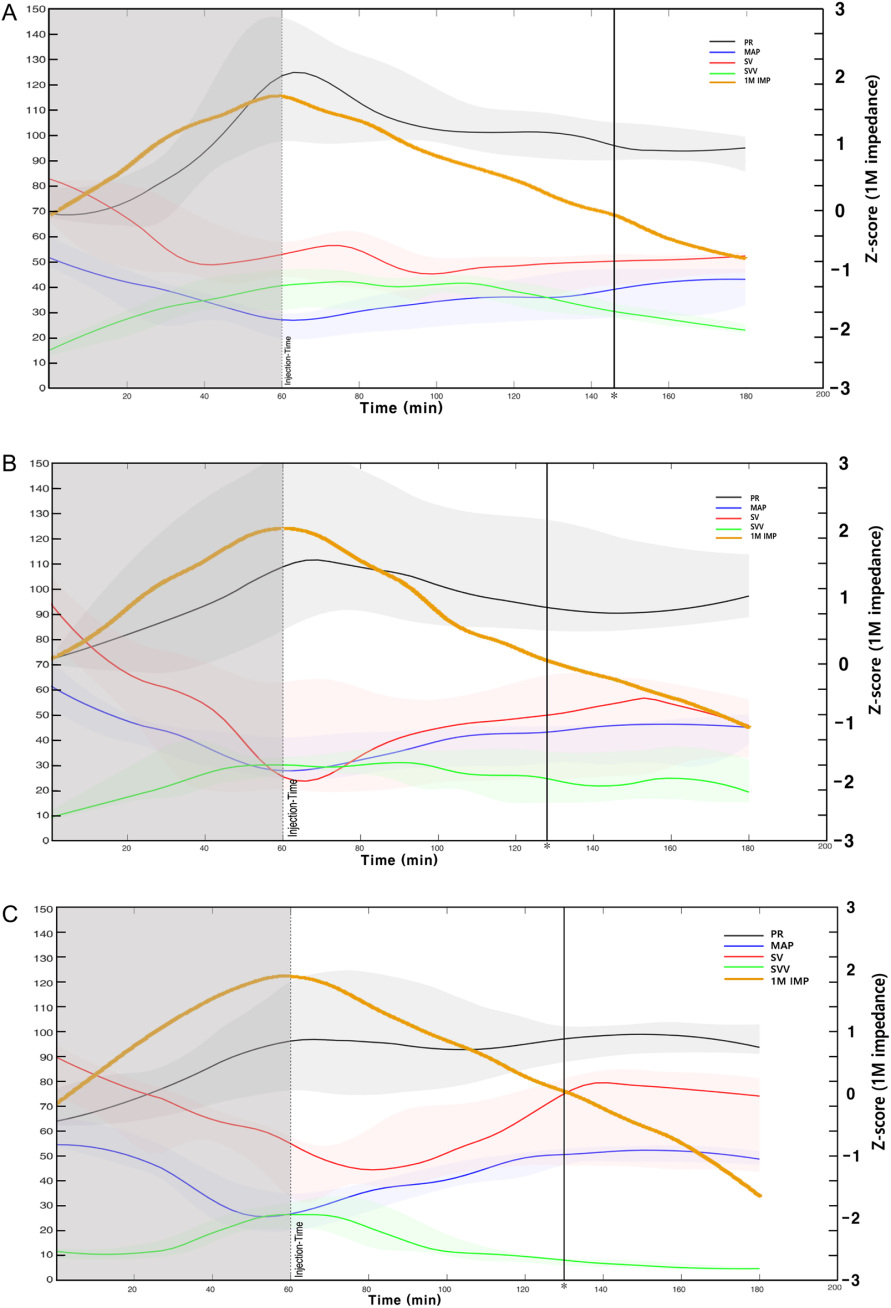
Results of hemodynamic monitoring over time. X = Time (minutes), Y (left) = PR, MAP, SV, SVV, Y (right) = Standardized value of 1M impedance. Standardized median curves, (black line – PR, blue line – MAP, red line – SV, green line – SVV, yellow line – 1M impedance), * of Figures (**A**–**C**) indicates 146, 128, 135 minutes, respectively. * of Figure (**C**) which indicates 128 minutes is 70 minutes after the start of fluid infusion, the time of a total of 1.2 L fluid infusion.

In all three groups, PR and SVV showed an increasing trend from the start of the experiment until the end of the controlled hemorrhage, while SV and MAP showed a decreasing trend (Fig. 6A–C). Additionally, PR and SVV tended to decrease from fluid infusion to the end of fluid infusion, while SV and MAP tended to increase (Fig. 6C). The increase in SV and MAP and decrease in PR and SVV according to the fluid infusion were significant in the combination of balanced crystalloid + 20% albumin. These trends were observed until approximately 70 min after fluid infusion and approximately 1.2 L of accumulated volume. After 70 min, no increase in MAP or SV was observed even with additional fluid infusion. The 1M impedance, which reflects total body water, increased from the start of the experiment until the end of the controlled hemorrhage in all three groups, and then decreased from the time of fluid infusion to the end of fluid infusion. MAP and SV did not increase after approximately 70 min after fluid infusion. This 70 min coincided with the time when the 1M impedance, which had increased after the start of controlled hemorrhage, recovered to its value at the starting point of the experiment (* of Fig. 6C).

**Figure 7:**
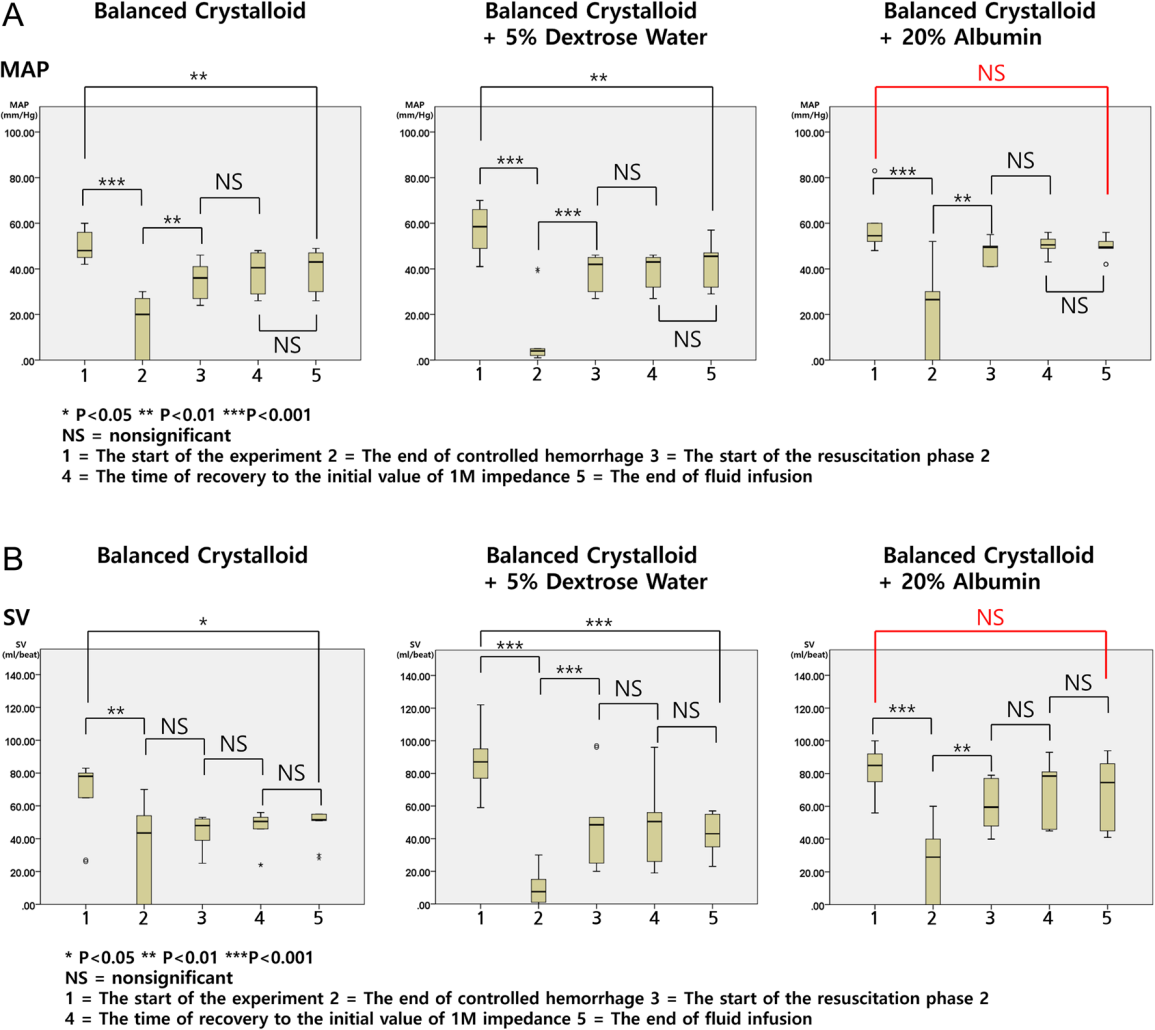
(**A**) Changes in MAP for each resuscitation fluid group at the start of the experiment, end of controlled hemorrhage, start of the resuscitation phase 2, time of recovery to the initial value of 1M impedance, and end of fluid infusion. (**B**) Changes in SV for each resuscitation fluid group at the start of the experiment, end of controlled hemorrhage, start of the resuscitation phase 2, time of recovery to the initial value of 1M impedance, and end of fluid infusion. *NS* nonsignificant.

On comparing MAP for each resuscitation fluid group at the start of the experiment, the end of controlled hemorrhage, the start of the resuscitation phase 2, the time of recovery to the initial value of 1M impedance, and the end of fluid infusion, the recovery of MAP to the point of before controlled hemorrhage was found only in the combination of balanced crystalloid + 20% albumin (Fig. 7A). In the balanced crystalloid group, MAP at the start of the experiment significantly decreased by 21.0% from 49.9 mmHg at the start of the experiment to 39.4 mmHg after the end of fluid infusion (p = 0.008; the start of the experiment—the end of controlled hemorrhage p < 0.001; the end of controlled hemorrhage—the start of the resuscitation phase 2 p = 0.002; the start of the resuscitation phase 2—the time of recovery to the initial value of 1M impedance p = 0.442). In the balanced crystalloid + 5% dextrose water group, MAP at the start of the experiment significantly decreased by 24.3% from 56.9 mmHg at the start of the experiment to 43.1 mmHg after the end of fluid infusion (p = 0.0067; the start of the experiment—the end of controlled hemorrhage p < 0.001; the end of controlled hemorrhage—the start of the resuscitation phase 2 p < 0.001; the start of the resuscitation phase 2—the time of recovery to the initial value of 1M impedance p = 0.892). In the balanced crystalloid + 20% albumin group, MAP at the start of the experiment decreased by 16.8% from 56.9 mmHg at the start of the experiment to 49.6 mmHg after the end of fluid infusion and was not statistically significant (p = 0.164; the start of the experiment—the end of controlled hemorrhage p < 0.001; the end of controlled hemorrhage—the start of the resuscitation phase 2 p = 0.002; the start of the resuscitation phase 2 – the time of recovery to the initial value of 1M impedance p = 0.189). In addition, MAP presented significant differences from the time of recovery to the initial value of 1M impedance to the end of fluid infusion in all three groups (p = 0.757, p = 0.284, p = 0.707, respectively).

On comparing the SV for each resuscitation fluid group at the start of the experiment, the end of controlled hemorrhage, the start of the resuscitation phase 2, the time of recovery to the initial value of 1M impedance, and the end of fluid infusion, the recovery of SV to the point of before controlled hemorrhage was also found only in the combination of balanced crystalloid + 20% albumin (Fig. 7B). In the balanced crystalloid group, the SV at the start of the experiment significantly decreased by 28.8% from 67.6 ml/beat at the start of the experiment to 48.1 ml/beat after the end of fluid infusion (p = 0.021; the start of the experiment—the end of controlled hemorrhage p = 0.009; the end of controlled hemorrhage—the start of the resuscitation phase 2 p = 0.331; the start of the resuscitation phase 2—the time of recovery to the initial value of 1M impedance p = 0.641). In the balanced crystalloid + 5% dextrose water group, the SV at the start of the experiment significantly decreased by 51.5% from 88 ml/beat at the start of the experiment to 42.7 ml/beat after the end of fluid infusion (p < 0.001; the start of the experiment—the end of controlled hemorrhage p < 0.001; the end of controlled hemorrhage—the start of the resuscitation phase 2 p < 0.001; the start of the resuscitation phase 2—the time of recovery to the initial value of 1M impedance p = 0.969). In the balanced crystalloid + 20% albumin group, the SV at the start of the experiment decreased by 17.4% from 81.4 ml/beat at the start of the experiment to 67.2 ml/beat after the end of fluid infusion, which was not statistically significant (p = 0.099; the start of the experiment—the end of controlled hemorrhage p < 0.001; the end of controlled hemorrhage—the start of the resuscitation phase 2 p = 0.001; the start of the resuscitation phase 2—the time of recovery to the initial value of 1M impedance p = 0.347). In addition, there was no significant difference in SV from the time of recovery to the initial value of 1M impedance to the end of fluid infusion in all three groups (p = 0.648, p = 0.527, p = 0.906, respectively).

## Discussion

### Evaluation of fluid responsiveness and potential use of BIA

In the results of this study (Fig. 7A and B), when fluid was infused with balanced crystalloid + 20% albumin, the point at which MAP and SV recovered to the values at the start of the experiment was the point at the end of resuscitation phase 1. Thereafter, no significant increase in MAP and SV was observed. Therefore, resuscitation phase 2 is considered to have contributed to unnecessary volume. The change in SV (ΔSV) is known to exhibit higher sensitivity and specificity for the determination of fluid responsiveness^40^. After a fluid challenge, when ΔSV is less than 10%, there is no fluid responsiveness^41^. In Fig. 7B, the SV at the end of resuscitation phase 1 and at the point when 1M impedance was recovered to the initial value was not statistically significantly different. Although not presented as numerical data in Fig. 7B, ΔSV of these two points is 9.1%, less than 10%. In Fig. 6C, MAP and SV did not increase after approximately 70 min after fluid infusion. This point coincided with the time when the 1M impedance, which had increased after the start of controlled hemorrhage, recovered to its value at the starting point of the experiment (* of Fig. 6C). This suggests the possibility of fluid responsiveness in hypovolemic shock patients based on the impedance changes measured with BIA. BIA was able to detect physiological changes during the resuscitation phase 2, when the changes of SV and MAP could not reflect physiological changes. The resuscitation phase 2 may be consistent with a more modest hypovolemia. Moreover, the ability of BIA to track fluid resuscitation in this phase may have utility.

### The importance of intracellular water and the meaning of 5% dextrose water

There is a study showed that cell shrinkage was a trigger of apoptotic signal transduction^42^. A typical example of a fluid to supply intracellular water is 5% dextrose water, which is redistributed into extracellular/intracellular water when intravenously infused. Dextrose water of 5% is technically an isotonic solution, but the glucose is rapidly metabolized by the liver and tissues, leaving only free water, making it a hypotonic solution of water^43,44^. Therefore, according to the water distribution in the body, when 1 L of 5% dextrose water is injected, 333 ml of the free water moves into extracellular water and 666 ml of the free water moves into intracellular water. Therefore, 5% dextrose water can be defined as a solution that helps supply intracellular water.

### Expected volume distribution after fluid infusion and the effect of albumin on the intracellular water movement

In Fig. 4–3, albumin theoretically distributes only in extracellular water; therefore, the graph was expected to show a straight line during fluid infusion after controlled hemorrhage. However, it showed a curved line. According to Haupt et al.^45^ several situations can increase vascular permeability, among which is shock status including trauma. In this increased vascular permeability status, resuscitation using colloids can lead to the movements of colloid particles toward interstitial space^45^. Ernest et al.^46^ infused normal saline or 5% albumin to septic shock patients and examined the volume distribution. The albumin leaked into the interstitial fluid after 5% albumin was infused. This led to an increase in interstitial osmotic pressure that was sufficient to draw intracellular water into extracellular water^46^. Therefore, the infusion of balanced crystalloid + 20% albumin, which was expected to only increase extracellular water, showed a change in intracellular water, which explains the curved shape in Fig. 4–3. During this experiment, the pigs’ urine output was measured but thought to be ignorable and excluded from the analysis (Supplement 2).

### Limitations

In a small animal study, we pragmatically choose both a standard volume and tested only 3 combinations of fluid types. These were done to align with general clinical practice options. Therefore, a follow-up study is necessary to determine the optimal combination ratio. This study was implemented on pigs, which may cause limitations in applying the results to humans. Although some studies use BIA and FloTrac in animal models, there is no clear evidence or guidelines for applying the two devices in these models. However, because of their similar physiology and anatomy to humans, pigs are considered to be ideal animal models for studying human health and diseases^47^. As the experiment was implemented on five pigs in each group, further research on a larger population of pigs or in humans would be necessary.

## Conclusions

Among the three types of fluid combinations, only balanced crystalloid + 20% albumin significantly restored MAP and SV to the same level as at the start of the experiment after the end of fluid infusion. Moreover, in the balanced crystalloid + 20% albumin group, the effect of the infused fluids on recovering the MAP and SV up to the recovery of the initial value of 1M impedance was the same as at the end of the fluid infusion. The change and the recovery of hemodynamic indices such as MAP and SV coincide with the change and the recovery of 1M impedance.

The results of this study suggest that using balanced crystalloid mixed with 20% albumin in hypovolemic shock-induced swine model may be helpful in securing hemodynamic stability, compared with balanced crystalloid single administration.

### Supplementary Information

Supplementary Information 1.

Supplementary Information 2.

## Data Availability

The data that support the findings of this study are available from Inbody co. but restrictions apply to the availability of these data, which were used under license for the current study, and so are not publicly available. Data are however available from the authors upon reasonable request and with permission of Inbody co. (Point of Contact: HyunJoo Shin, Position: BWA part manager, Email: hyunju416@inbody.com).
