# Supplementary material for: Analysis of hemodynamics and impedance using bioelectrical impedance analysis in hypovolemic shock-induced swine model
Source: Sci Rep. 2024 Jul 2;14:15077. doi: 10.1038/s41598-024-65847-y (PMC11219720; doi:10.1038/s41598-024-65847-y)
Supplement: Supplementary file 1 — Supplementary Information 1. [file 41598_2024_65847_MOESM1_ESM.docx]

**Supplement 1. The data on average proportion of blood loss per body weight and body surface area (BSA)**

| **Group** | **Numbers** | **Blood**  **Loss**  **(L)** | **BSA (m^2^)** | **Blood loss/BSA**  **(L/m^2^)** | **Kruskal-Wallis test** | **Numbers** | **Blood**  **loss**  **(L)** | **Body**  **weight**  **(kg)** | **Blood loss/Body weight (L/kg)** | **Kruskal-Wallis test** |
| --- | --- | --- | --- | --- | --- | --- | --- | --- | --- | --- |
| **Balanced crystalloid 2 L**  **(A)** | **1** | **1** | **1.03** | **0.97** | **p = 0.85**  **post-hoc analysis**  **(Mann-Whitney U test)**  **Group (A) – (B): p = 0.92**  **Group (B) – (C): p = 1**  **Group (C) – (A): p = 0.53**  **No significant differences among the three groups in Blood loss/BSA** | **1** | **1** | **38.9** | **0.0257** | **p = 0.86**  **post-hoc analysis**  **(Mann-Whitney U test)**  **Group (A) – (B): p = 0.92**  **Group (B) – (C): p = 0.84**  **Group (C) – (A): p = 0.55**  **No significant differences among the three groups in Blood loss/Body weight** |
|  | **2** | **1** | **0.99** | **1.01** |  | **2** | **1** | **36** | **0.0278** |  |
|  | **3** | **1** | **1.03** | **0.97** |  | **3** | **1** | **39** | **0.0256** |  |
|  | **4** | **1** | **1.09** | **0.92** |  | **4** | **1** | **44.7** | **0.0224** |  |
|  | **5** | **1** | **0.95** | **1.05** |  | **5** | **1** | **32** | **0.0313** |  |
| **Balanced crystalloid 1 L + 5% Dextrose Water 1 L**  **(B)** | **1** | **1** | **1** | **1.00** |  | **1** | **1** | **36.2** | **0.0276** |  |
|  | **2** | **1** | **0.98** | **1.02** |  | **2** | **1** | **34.6** | **0.0289** |  |
|  | **3** | **1** | **1.04** | **0.96** |  | **3** | **1** | **39.8** | **0.0251** |  |
|  | **4** | **1** | **1.07** | **0.93** |  | **4** | **1** | **43.1** | **0.0232** |  |
|  | **5** | **1** | **0.95** | **1.05** |  | **5** | **1** | **32** | **0.0313** |  |
| **Balanced crystalloid 1.6 L**  **+ 20% Albumin 400 ml**  **(C)** | **1** | **1** | **1.01** | **0.99** |  | **1** | **1** | **37.2** | **0.0269** |  |
|  | **2** | **1** | **1.01** | **0.99** |  | **2** | **1** | **37.4** | **0.0267** |  |
|  | **3** | **1** | **1.02** | **0.98** |  | **3** | **1** | **38.4** | **0.0260** |  |
|  | **4** | **1** | **0.98** | **1.02** |  | **4** | **1** | **34.9** | **0.0287** |  |
|  | **5** | **1** | **1** | **1.00** |  | **5** | **1** | **36.8** | **0.0272** |  |
