# Supplementary material for: Analysis of hemodynamics and impedance using bioelectrical impedance analysis in hypovolemic shock-induced swine model
Source: Sci Rep. 2024 Jul 2;14:15077. doi: 10.1038/s41598-024-65847-y (PMC11219720; doi:10.1038/s41598-024-65847-y)
Supplement: Supplementary file 2 — Supplementary Information 2. [file 41598_2024_65847_MOESM2_ESM.docx]

**Supplement 2. Urine output and body temperature of the experimental animals**

|  | **Urine**  **output**  **(cc/hr)** | **Experimental phase** | | | **Body**  **temperature^a^**  **(︒C)** | **Experimental phase** | | | |
| --- | --- | --- | --- | --- | --- | --- | --- | --- | --- |
| **Time elapsed** |  | **60 mins** | **60 mins** | **60 mins** |  | **0 min** | **60 mins** | **60 mins** | **60 mins** |
| **Fluids** | **Numbers** | **Controlled**  **hemorrhage** | **Resuscitation**  **phase 1** | **Resuscitation**  **phase 2** | **Numbers** | **Initial** | **Controlled**  **hemorrhage** | **Resuscitation**  **phase 1** | **Resuscitation**  **phase 2** |
| **Balanced crystalloid 2 L** | **1** | **20** | **10** | **10** | **1** | **38** | **38.2** | **38** | **38** |
|  | **2** | **30** | **20** | **10** | **2** | **37** | **37.6** | **37.1** | **36.6** |
|  | **3** | **20** | **10** | **40** | **3** | **38.1** | **38.3** | **38** | **37.6** |
|  | **4** | **10** | **20** | **10** | **4** | **37.7** | **37.9** | **37.3** | **36.9** |
|  | **5** | **50** | **20** | **40** | **5** | **36.7** | **37** | **36.6** | **36.5** |
| **Balanced crystalloid 1 L + 5% Dextrose Water 1 L** | **1** | **80** | **30** | **20** | **1** | **37.6** | **37.5** | **36.9** | **36.8** |
|  | **2** | **10** | **20** | **10** | **2** | **37.2** | **36.4** | **36.3** | **36.1** |
|  | **3** | **30** | **20** | **10** | **3** | **39.2** | **39.7** | **39.3** | **38.9** |
|  | **4** | **15** | **10** | **30** | **4** | **38** | **38** | **37.5** | **37.1** |
|  | **5** | **0** | **0** | **0** | **5** | **36.5** | **36.8** | **36.2** | **35.8** |
| **Balanced crystalloid 1.6 L + 20% Albumin 400 ml** | **1** | **10** | **0** | **0** | **1** | **37.3** | **36.2** | **35.1** | **34.5** |
|  | **2** | **80** | **30** | **120** | **2** | **37** | **36.5** | **35.9** | **35.4** |
|  | **3** | **130** | **10** | **40** | **3** | **36.8** | **37** | **36.9** | **36.7** |
|  | **4** | **50** | **30** | **60** | **4** | **39** | **39.3** | **38.5** | **38** |
|  | **5** | **0** | **20** | **90** | **5** | **36** | **36.2** | **35.5** | **35.1** |

**a) Measured with a rectal thermometer**
